# A randomized trial of ‘fresh start’ text messaging to improve return to care in people with HIV who missed appointments in South Africa

**DOI:** 10.1097/QAD.0000000000003939

**Published:** 2024-06-10

**Authors:** Christine Njuguna, Lawrence Long, Preethi Mistri, Candice Chetty-Makkan, Brendan Maughan-Brown, Alison Buttenheim, Laura Schmucker, Sophie Pascoe, Harsha Thirumurthy, Cara O’Connor, Chipo Mutyambizi, Barry Mutasa, Kate Rees

**Affiliations:** a Anova Health Institute, Parktown, Johannesburg, South Africa; b Department of Global Health, Boston University School of Public Health, Boston, MA, USA; c Health Economics and Epidemiology Research Office, Faculty of Health Sciences, University of the Witwatersrand, Johannesburg; d Southern Africa Labour and Development Research Unit, University of Cape Town, Cape Town, South Africa; e Department of Family and Community Health, School of Nursing, University of Pennsylvania; f Department of Medical Ethics and Health Policy, Perelman School of Medicine, University of Pennsylvania, Philadelphia, PA, USA; g Department of Community Health, School of Public Health, Faculty of Health Sciences, University of Witwatersrand, Johannesburg, South Africa.

**Keywords:** ART re-engagement, behavioural science, fresh start, message framing, randomised controlled trial, retention in care, text messaging

## Abstract

**Objective:**

Treatment interruptions are a barrier to successful antiretroviral therapy (ART). ‘Fresh start messages’, which leverage significant days on the calendar (e.g., new year, public holiday) in order to prompt action, have the potential to encourage people with HIV (PWH) to return to care. We evaluated a ‘fresh start’ intervention (text messages) to increase return to care in PWH who had missed their last appointment.

**Design:**

A three arm 1 : 1:1 individual randomised controlled trial.

**Methods:**

We randomized adults in Capricorn District who had missed ART appointments by >28 days to: no text message; *unframed* messages (fresh start not mentioned); or *framed* messages (fresh start mentioned). Randomization was stratified by treatment interruption duration and across two holidays (Youth Day, Mandela Day). The primary outcome was an ART-related clinic visit at ≤45 days of the first message.

**Results:**

9143 participants were randomised. For Youth Day, 1474 and 1468 were sent *unframed* and *framed* messages respectively, with 13.4% sent these messages having an ART visit vs. 11.9% not sent a message [adjusted odds ratio (aOR) 1.2; 95% confidence interval (CI): 1.0–1.4, *P*-value = 0.075]. For Mandela Day, 1336 and 1334 were sent *unframed* and *framed* messages respectively, with 6.7% sent these messages having an ART-related clinic visit vs. 5.4% not sent a message (aOR 1.2; 95% CI: 1.0–1.6; *P*-value = 0.100).

**Conclusions:**

Low-cost text messages sent around a ‘fresh start’ date may increase the likelihood that patients who miss appointments return to care. This study suggests the potential of text messaging for motivating return to care.

## Introduction

South Africa has the largest antiretroviral therapy (ART) programme in the world, with an estimated 5.7 million people on ART in 2022 [1]. Despite this, the country is not attaining sufficient ART coverage and viral suppression, the second and third of the UNAIDS 95–95–95 targets [2]. Projections show that by 2030, 96% of people with HIV (PWH) will know their status, but of these only 78% will be on ART, and among those on ART, 91% will be virally suppressed [2]. Treatment interruptions are a contributing factor [2]. Disengagement from care is associated with an increased risk of drug resistance, onward HIV transmission, opportunistic infections, and mortality [3]. Data from South African cohorts of PWH have reported ART treatment interruption and/or loss to follow up (LTFU) ranging from 18% to 30% [3–6].

South African guidelines recommend telephonic contact and home visits to encourage PWH to re-engage with care after they have missed an appointment. If facilities are unable to locate a client after 3 months, they are classified as disengaged and further tracing is stopped [7]. Text messaging is not part of national guidelines on tracing but is an inexpensive and easily accessible intervention that has previously been used to promote adherence [8], appointment keeping [9], and re-engaging PWH [10]. The content of text messages may influence their overall effectiveness.

The ‘fresh start’ effect leverages moments in time (temporal landmarks) when an individual may be open to change. It relates to the idea of ‘new beginnings’ and utilises the importance that people place on specific moments to motivate changes in behaviour [11,12]. Temporal landmarks include the start of new time periods (year, month, week) as well as religious or cultural holidays, and serve as an opportunity to leave the ‘imperfect self’ in the past and aspire to an improved ‘future self’ [11,13]. The fresh start effect has been reported to increase the likelihood of individuals engaging in their personal goals, including health goals [13]. While ‘fresh start’ interventions hold promise, fresh start messaging to improve HIV treatment outcomes has not been evaluated.

## Methods

### Study design and setting

We conducted an individual-level randomised controlled trial (RCT) – following the Consolidated Standards of Reporting Trials (CONSORT) guidelines [14] in Capricorn District, Limpopo Province, South Africa [15]. HIV prevalence among people aged 15–49 years in the province is estimated at 17.2% [16]. The Department of Health has classified 24 public-sector clinics with a high number of people on ART as priority clinics.

### Study population

Data was extracted from TIER.Net, an electronic database documenting demographic characteristics, clinical interactions, and treatment outcomes of PWH in South Africa. Our study included all adult clients (≥18 years) with a documented mobile phone number who had missed their last scheduled clinic appointment by >28 days. Clients known to be deceased or transferred to another clinic were excluded.

### Randomization

Eligible clients were randomized into one of three arms using computer-generated random numbers at a 1 : 1:1 allocation: standard of care (SOC) arm who were not sent any text messages; *unframed* text message arm; and *framed* text message arm. We also stratified the sample based on duration of treatment interruption (<6 months or ≥6 months), and temporal landmarks (Youth Day and Mandela Day).

### Intervention

We developed text messages that harnessed the fresh start effect and evaluated their impact on re-engagement among PWH who had missed their ART appointment. We used two temporal landmarks, Youth Day, a public holiday (Thursday, 16 June 2022), and Mandela Day, Nelson Mandela's birthday (Monday, 18 July 2022).

Anova Health Institute, the Capricorn District PEPFAR/USAID implementing partner, delivered the interventions through a bulk text messaging service. Messages were developed by a panel of ART programme and behavioural economics experts and built upon the National Department of Health (NDOH) ART re-initiation ‘Welcome Back’ campaign*. Framed* text messages integrated fresh start messaging and were piloted with site programme teams. All text messages were sent in English.

### Arm 1: standard of care – no text message

The SOC arm included routine tracing activities that are part of existing HIV programmes regardless of trial processes. The fidelity of SOC follow-up activities could not be monitored. ART clients who failed to return to the health clinic for scheduled appointments were identified within seven days. As per current NDOH guidelines, three telephone attempts are made within 14 days. If unsuccessful, records are checked for additional contact numbers [17]. Thereafter, Ward-based Primary Healthcare Outreach Teams conduct a home visit.

### Arm 2: *unframed* text message

In addition to the SOC [7], this arm received two text messages encouraging return to the clinic. The first text message was sent five days before each temporal landmark (Youth Day and Mandela Day) and the second the day after the temporal landmark. This message did not explicitly refer to the temporal landmarks or the fresh start effect. Text message content in this arm had a Flesch-Kincaid readability level of sixth grade (easy to read). [18] The first unframed message read:

Hello! This is a message from your clinic. We invite you to return to clinic soon, we missed you at your last appointment. Check in at Reception, we are ready to help. Welcome Back!

### Arm 3: *framed* text message

In addition to the SOC [7], this arm received two text messages encouraging return to the clinic. Messages were also sent 5 days before and one day after the temporal landmark. The *framed* messages specifically mentioned the temporal landmark and referred to a ‘new beginning.’ Message testing revealed that a ‘new beginning’ was easier to understand than a ‘fresh start.’ Messages had a readability level of sixth grade (easy to read). The first framed message read:

Happy Youth Day for 16 June! We invite you to return to your clinic soon, we missed you at your last appointment. Your new beginning starts at Reception – we are ready to help. Welcome Back!

See Table 1, Supplemental Digital Content for all text messages.

### Primary outcome

The primary outcome was whether the participant returned for an ART clinic visit within 45 days of the first text message for both intention-to- treat (ITT) and per-protocol analysis (restricted to delivered text messages). Outcome ascertainment was measured as the difference between the ART visit date and the date of the first text message for all arms.

### Sample size calculation

Based on programme data, we estimated a baseline return rate of 3%, and anticipated an increase of two percentage points. The sample size was estimated at 1605 participants using a significance level of 0.05, power of 0.8 and an effect size difference of 2%. The total sample size was 9630 (1605 × 3 arms × 2 strata). The actual sample was 4103 in the <6 months stratification and 5227 in the ≥6 months stratification due to a smaller number of people with a missed appointment of <6 months on TIER.Net.

### Data analysis

Analysis was conducted in STATA 18 (Stata Corp, Texas). Characteristics between study arms were assessed using chi-squared tests. Multivariable logistic regressions were conducted to determine the impact of the intervention on return to care, adjusting for age at randomization, sex, ART duration, duration of treatment interruption, enrolment into differentiated ART pick-up, sub-district, and priority clinic. These variables were selected a priori as they have been associated with return to care and were available on TIER.Net.

Preregistered analyses assessed the impact of any text message (*unframed* and *framed*) compared to no text message, and whether *unframed* text messages had a different impact than *framed* text messages. These were conducted separately for Youth Day and Mandela Day and by treatment interruption duration (<6 months and ≥6 months).

Randomisation was done on 10 June. For participants who received a text message on Youth Day (16 June), there were two days between randomisation and first message. However, for Mandela Day (18 July), there had been 31 days since randomization. We used the same individuals for the SOC arm for Youth Day and Mandela Day. However, we calculated their outcomes twice, once with Youth Day and once with Mandela Day timing. Therefore, for the SOC arm (and intervention arms), the time between intervention and outcome ascertainment was 45 days for both temporal landmarks.

### Ethical approval and trial registration

Ethical approval was provided by the University of Witwatersrand Human Research Ethics Committee (HREC:220207), the University of Pennsylvania Institutional Review Board (IRB:851123), Boston University IRB (H-42789), and the Limpopo Province Research Committee. No informed consent was obtained, as per HREC waiver.

This RCT was registered with the Pan African Clinical Trial Registry (www.pactr.org), registration number – PACTR202202748760768 and the South African National Clinical Trial Registry (https://sanctr.samrc.ac.za/), registration number – DOH-27-042022-6703.

## Results

**Fig. 1:**
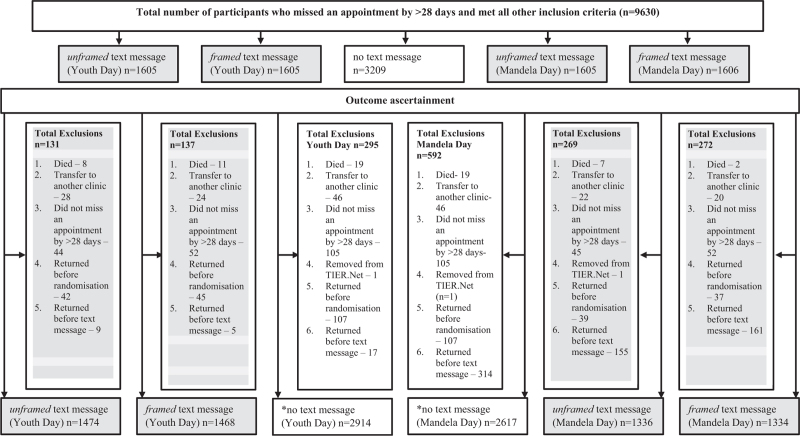
Study consort. There was only one no text message arm, and the same people were used for Youth Day and Mandela Day.

At the time of data extraction, 9630 participants met the inclusion criteria. Combining both treatment interruption stratifications, participants were randomized into three arms: no text message (*n* = 3209), *framed* text message (*n* = 3211), and *unframed* text message (*n* = 3210) **(**Fig. 1**).** Within the *framed* and *unframed* text message arms, participants were randomly allocated into two groups: *framed* Youth Day (*n* = 1605), *framed* Mandela Day (*n* = 1606), *unframed* Youth Day (*n* = 1605), and *unframed* Mandela Day (*n* = 1605). Text message delivery rates ranged from 51% to 56% and did not differ between arms, however, they did differ by treatment interruption stratification, at 57–62% for < 6 months and 48–53% for ≥6 months.

The outcome, ART visit within 45 days of first message, was ascertained at two time points, Youth Day and Mandela Day. Following outcome ascertainment, 563 were excluded from the Youth Day analysis (131 unframed, 137 framed, 295 no text message) and 1133 from the Mandela Day analysis (269 unframed, 272 framed, 592 no text message). Major reasons for exclusion were clients who returned to the clinic before the message was sent (225/563, 40.0% Youth Day and 813/1133, 71.8% Mandela Day), clients who had not missed an appointment by >28 days (201/563, 35.7% Youth Day and 202/1133, 17.8% Mandela Day) and recorded transfer outs (98/563, 17.4% Youth Day and 88/1133, 7.8% Mandela Day). 5856 and 5287 participants were included in the final Youth Day and Mandela Day analyses (Fig. 1).

### Baseline characteristics

All demographic and clinical characteristics were similar across study arms (Table 1). Most participants were female (69% Youth Day and 63% Mandela Day), aged 25 to 49 years (77% Youth Day and 70% Mandela Day), on ART for ≥12 months (61% Youth Day and 53% Mandela Day).

**Table 1:** Characteristics of participants in the study arms.

Participant characteristics	No text message – Youth Day (*n* = 2914)	*Unframed* Youth Day text message (*n* = 1474)	*Framed* Youth Day text message (*n* = 1468)	No text message – Mandela Day (*n* = 2617)	*Unframed* Mandela Day text message (*n* = 1336)	*Framed* Mandela Day text message (*n* = 1334)
Age at randomisation (years)						
18–24	180 (6.18%)	77 (5.22%)	94 (6.40%)	163 (6.23%)	89 (6.66%)	82 (6.15%)
25–49	2236 (76.73%)	1146 (77.75%)	1132 (77.11%)	2022 (77.26%)	1045 (78.22%)	1021 (76.54%)
≥50	498 (17.09%)	251 (17.03%)	242 (16.49%)	432 (16.51%)	202 (15.12%)	231 (17.32%)
Sex						
Male	908 (31.16%)	465 (31.55%)	463 (31.54%)	809 (30.91%)	399 (29.87%)	419 (31.41%)
Female	2006 (68.84%)	1009 (68.45%)	1005 (68.46%)	1808 (69.09%)	937 (70.13%)	915 (68.59%)
ART duration^a^ (months)						
<6	815 (27.98%)	416 (28.24%)	431 (29.36%)	789 (30.16%)	426 (31.91%)	409 (30.71%)
6–12	306 (10.50%)	145 (9.84%)	166 (11.31%)	286 (10.93%)	141 (10.56%)	146 (10.96%)
>12	1792 (61.52%)	912 (61.91%)	871 (59.33%)	1541 (58.91%)	768 (57.53%)	777 (58.33%)
Treatment interruption stratification (months)						
<6	1093 (37.51%)	564 (38.26%)	554 (37.74%)	812 (31.03%)	431 (32.26%)	430 (32.23%)
≥6	1821 (62.49%)	910 (61.74%)	914 (62.26%)	1805 (68.97%)	905 (67.74%)	904 (67.77%)
Enrolled in differentiated care						
No	2377 (81.57%)	1211 (82.16%)	1216 (82.83%)	2198 (83.99%)	1105 (82.71%)	1111 (83.28%)
Yes	537 (18.43%)	263 (17.84%)	252 (17.17%)	419 (16.01%)	231 (17.29%)	223 (16.72%)
Priority clinic						
No	1288 (44.20%)	671 (45.52%)	652 (44.41%)	1108 (42.34%)	617 (46.18%)	613 (45.95%)
Yes	1626 (55.80%)	803 (54.48%)	816 (55.59%)	1509 (57.66%)	719 (53.82%)	721 (54.05%)
Sub-district						
Blouberg	340 (11.67%)	184 (12.48%)	155 (10.56%)	300 (11.46%)	127 (9.51%)	157 (11.77%)
Lepelle-Nkumpi	527 (18.09%)	267 (18.11%)	269 (18.32%)	459 (17.54%)	258 (19.31%)	267 (20.01%)
Molemole	282 (9.68%)	138 (9.36%)	134 (9.13%)	257 (9.82%)	135 (10.10%)	129 (9.67%)
Polokwane	1765 (60.57%)	885 (60.04%)	910 (61.99%)	1601 (61.18%	816 (61.08%)	781 (58.55%)

^a^ Excludes one participant from the No text message-Youth Day with missing information on ART duration, one participant from the *unframed* Youth Day text message, one participant from the No text message-Mandela Day, one participant from the *unframed* Mandela Day text message, and two participants from the *framed* Mandela Day text message arm. ^∗^All *P*-values testing the differences between the No text message arms and the text message arms were >0.05 for all variables except for priority clinic, where the *P*-value testing the difference between the No text message-Mandela arm and the unframed Mandela Day text message and framed Mandela Day text message was 0.021 and 0.030, respectively.

### Intervention effect on ART visit

#### Youth Day

ART visits within 45 days occurred among 13.4% (95% confidence interval (CI): 12.2–14.6); 393/2942 of those sent any message compared to 11. 9% (95% CI: 10.7– 13.1); 346/2914 not sent a message (difference: 1.5%, *P* = 0.087). In per-protocol analysis, ART visits within 45 days occurred among 16.6% (95% CI: 14.7–18.6); 240/1449 receiving any message compared to 11.9% in the no message group (difference: 4.7%; *P* < 0.00) (Table 2). In multivariable analysis, participants sent any message had increased odds of an ART visit [adjusted odds ratio (aOR): 1.2; 95% CI: 1.0–1.4] compared to no message (Table 3).

**Table 2:** Proportion of participants returning for an ART visit in intention to treat analysis and per protocol analysis.

	Youth Day				Mandela Day			
Text message arm	Sample size	ART visit % (95% CI)	% difference	*P*-value	Sample size	ART visit % (95% CI)	% difference	*P*-value
All participants								
No text message	2914	11.87% (10.72–13.10)	1.49%	0.087	2617	5.39% (4.55–6.32)	1.35%	0.039
Any text message (*framed + unframed*)	2942	13.36% (12.15–14.64)			2670	6.74% (5.82–7.76)		
Any text message^∗^ (delivered only)	1449	16.56% (14.68–18.58)	4.69%	0.000	1331	8.56% (7.12–10.20)	3.17%	0.000
*Unframed* text message	1474	14.45% (12.69–16.35)	2.19%	0.081	1336	6.81% (5.52–8.30)	0.14%	0.886
*Framed* text message	1468	12.26% (10.63–14.05)			1334	6.67% (5.39–8.15)		
Treatment interruption <6 months								
No text message	1093	29.46% (26.77–32.26)	3.10%	0.115	812	13.79% (11.49–16.36)	2.47%	0.159
Any text message (*framed* + *unframed*)	1118	32.56% (29.82–35.39)			861	16.26% (13.86–18.90)		
Any text message^∗^ (delivered only)	617	35.33% (31.56–39.25)	5.87%	0.012	462	19.26% (15.77–23.16)	5.47%	0.010
*Unframed* text message	564	35.82% (31.85–39.93)	6.58%	0.019	431	17.40% (13.94–21.32)	2.28%	0.364
*Framed* text message	554	29.24% (25.48–33.22)			430	15.12% (11.86–18.86)		
Treatment interruption ≥6 months								
No text message	1821	1.32% (0.85–1.95)	0.27%	0.493	1805	1.61% (1.08–2.30)	0.60%	0.184
Any text message (*framed* + *unframed*)	1824	1.59% (1.07–2.28)			1809	2.21% (1.58–3.00)		
Any text message^∗^ (delivered only)	832	2.64% (1.66–3.98)	1.32%	0.015	869	2.88% (1.87–4.22)	1.27%	0.029
*Unframed* text message	910	1.21% (0.60–2.15)	0.76%	0.194	905	1.77% (1.01–2.86)	0.88%	0.200
*Framed* text message	914	1.97% (1.17–3.09)			904	2.65% (1.71–3.92)		

∗ Any text message-*unframed* text message and *framed* text message.

**Table 3:** Multivariable logistic regression of the association between predictor variables and ART visit outcome in participants randomized to no text message and any text message arms for Youth Day and Mandela Day temporal landmarks.

	Youth Day text messages (*n* = 5854)						Mandela Day text messages (*n* = 5283)					
Variables	Unadjusted odds ratio	95% CI	*P*-value	Adjusted odds ratio	95% CI	*P*-value	Unadjusted odd ratio	95% CI	*P*-value	Adjusted odds ratio	95% CI	*P*-value
Text message arm												
No text message	Ref			Ref			Ref			Ref		
Any text message^∗^	1.14	0.98–1.34	0.087	1.17	0.98–1.40	0.075	1.27	1.01–1.59	0.040	1.22	0.96–1.55	0.100
Age at randomization (years)												
18–24	Ref			Ref			Ref			Ref		
25–49	0.96	0.69–1.35	0.821	1.14	0.78–1.65	0.499	0.62	0.42–0.93	0.021	0.72	0.47–1.10	0.132
≥50	1.58	1.10–2.26	0.013	1.65	1.09–2.49	0.017	0.84	0.53–1.33	0.457	0.83	0.50–1.36	0.457
Sex												
Male	Ref			Ref			Ref			Ref		
Female	0.92	0.78–1.09	0.338	1.03	0.85–1.25	0.756	1.13	0.88–1.45	0.332	1.23	0.94–1.60	0.130
ART duration (months)												
<6	Ref			Ref			Ref			Ref		
6–12	2.51	1.68–3.75	0.000	2.43	1.58–3.74	0.000	0.99	0.54–1.84	0.979	0.89	0.47–1.66	0.708
>12	6.46	4.86–8.58	0.000	3.21	2.35–4.38	0.000	3.75	2.68–5.26	0.000	1.96	1.36–2.83	0.000
Treatment interruption (months)												
<6	Ref			Ref			Ref			Ref		
≥6	0.03	0.02–0.04	0.000	0.04	0.03–0.05	0.000	0.11	0.08–0.14	0.000	0.14	0.11–0.19	0.000
Enrolled in differentiated care												
No	Ref			Ref			Ref			Ref		
Yes	3.19	2.70–3.78	0.000	1.17	0.96–1.43	0.114	3.44	2.71–4.37	0.000	1.59	1.21–2.10	0.001
Priority clinic												
No	Ref			Ref			Ref			Ref		
Yes	0.56	0.47–0.65	0.000	0.72	0.60–0.87	0.001	0.58	0.46–0.73	0.000	0.71	0.54–0.92	0.010
Sub-district												
Blouberg	Ref			Ref			Ref			Ref		
Lepelle-Nkumpi	1.01	0.77–1.33	0.938	1.13	0.83–1.54	0.449	1.05	0.71–1.55	0.794	1.16	0.77–1.75	0.479
Molemole	0.72	0.51–1.01	0.055	0.77	0.52–1.13	0.186	0.66	0.40–1.09	0.106	0.77	0.46–1.31	0.337
Polokwane	0.77	0.61–0.98	0.033	0.88	0.67–1.17	0.392	0.73	0.52–1.04	0.078	0.84	0.57–1.23	0.360

∗ Any text message – *framed* and *unframed t*ext message.

ART visits occurred among 14.5% (95% CI: 12.7–16.4); 213/1474, in the *unframed* message arm compared to 12.3% (95% CI: 10.6–14.1); 180/1468 in the *framed* message arm (difference: 2.2%; *P* = 0.081) (Table 2).

#### Mandela Day

ART visits occurred among 6.7% (95% CI: 5.8–7.8); 180/2670 of those sent any message compared to 5.4% (95% CI:4.6–6.3); 141/2617 not sent a message (difference 1.4%; *P* = 0.039). In per-protocol analysis, ART visits occurred among 8.6% (95% CI: 7.1–10.2); 114/ 1331 of those receiving any message compared to 5.4% not sent a text message (difference: 3.2%; *P* < 0.00) (Table 2). A message was associated with increased odds of return (aOR 1.2; 95% CI: 1.0–1.6) vs. no message (Table 3).

ART visits occurred in 6.8% (95% CI: 5.5–8.3); 91/1336 in the *unframed* text message arm compared to 6.7% (95% CI: 5.4–8.2); 89/1334 in the *framed* text message arm (difference: 0.1%; *P* = 0.886) (Table 2).

#### Sub-group analysis

**Fig. 2:**
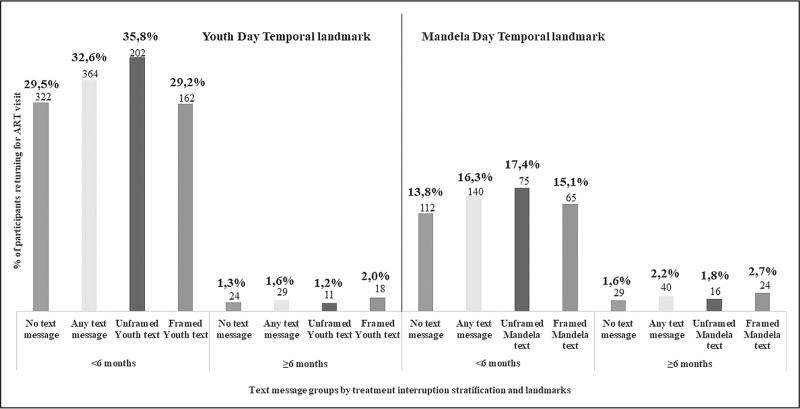
Proportions of participants with an ART visit by text message arm and treatment interruption stratification for Youth Day and Mandela Day temporal landmarks.

In sub-group analysis, ART visits were more likely among participants with treatment interruption <6 months compared to ≥6 months across all message groups (Fig. 2).

For Youth Day, treatment interruption stratification <6 months, 32.6% (95% CI: 29.8–35.4); 364/1118 sent any messages compared to 29.5% (95% CI: 26.8–32.3); 322/1093 not sent messages had an ART visit (difference 3.1%; *P* = 0.115) (Table 2). In per-protocol analysis, 35.3% (218/617; 95% CI: 31.6–39.3) of those receiving any message compared to 29.5% of those not sent messages had an ART visit (difference: 5.9%; *P* = 0.012).

In the treatment interruption stratification ≥6 months, 1.6% (29/1824; 95% CI: 1.1–2.3); sent any message compared to 1.3% (24/1821; 95% CI:0.9–2.0); not sent a message had an ART visit (difference: 0.3%; *P* = 0.493). In per-protocol analysis, 2.6% (22/832; 95% CI: 1.7–4.0); of those receiving any message compared to 1.3% of those not sent a message had an ART visit (difference: 1.3%; *P* = 0.015). Sub-group analysis for Mandela Day showed similar patterns, see Table 2.

There were no significant associations between any message and return to care in either stratum in multivariable analysis for Youth Day (Table 2, Supplemental Digital Content) or Mandela Day (Table 3, Supplemental Digital Content).

### Other factors associated with ART visits

In multivariable logistic regression for Youth Day, being on ART for ≥6 months at the time of the missed appointment increased odds of an ART visit compared to those <6 months on ART (Table 3). Decreased odds of an ART visit were seen among those with a treatment interruption ≥6 months (aOR: 0.04; 95% CI: 0.0–0.1), and those enrolled in a priority clinic (aOR 0.7; 95% CI: 0.6–0.9) (Table 3). We observed similar associations for Mandela Day.

### Ancillary analyses

We conducted two analyses to explore differences between message type and return to care. The first compared *framed* messages vs. no messages and *unframed* messages vs. no messages. There was no difference in ART visits between the *framed* messages vs. no text messages for either landmark. However, an *unframed* Youth Day message was associated with increased odds of return to care compared to no message (aOR 1.3; 95% CI: 1.0–1.6) (Table 4, Supplemental Digital Content) The second analysis compared *framed* to *unframed* text messages and found no difference in outcomes for either landmark (Table 4, Supplemental Digital Content).

## Discussion

This study demonstrated the potential of text messages leveraging the ‘fresh start’ effect to encourage return to care among PWH in South Africa. A higher proportion of participants sent any text message (*framed* and *unframed*) returned to care compared to participants sent no text message, although the difference was not significant for Youth Day. The increase in return rates was small, but was achieved with very low-cost, and warrants further exploration.

This is the first published data from South Africa reporting on fresh start text messaging to re-engage PWH who have disengaged from care. Most studies on text messaging for improving retention have targeted engaged PWH [19,20] and have seldom used behavioural science principles such as the ‘fresh start’ effect. Text messages have previously been used to re-engage PWH outside South Africa, with some studies reporting increased return to care [10], including studies from high income countries [21].

Our data highlights the value of text messaging as a re-engagement intervention, in addition to existing client tracing. Increases in return to care after sending a text message were small (1.5% for Youth Day and 1.4% for Mandela Day). However, when restricted to those who received text messages (per-protocol analysis), we reported bigger differences, 4.7% and 3.2%. In ITT and per-protocol analysis, the greatest benefit was observed in those who had missed appointments by less than six months. This finding suggests that text messaging aimed at re-engaging ART clients should be targeted to those who have missed appointments more recently. Text messages are an inexpensive intervention [8], and could be easily implemented at scale and integrated into existing bulk messaging. The cost of sending messages in this study was R0.21/∼US$0.01 per message, or R0.83/∼US$0.05 per participant.

Based on programme data of text messages for appointment reminders, we anticipated a delivery rate of about 70%. However, delivery rates were lower (<60%). Not updating contact details, and poor cellular network coverage in more rural areas could have contributed to lower delivery rates. These factors may also explain the relatively small effect sizes and should be considered in future interventions. Return to care was higher in those who received a text message, suggesting that successful delivery has a beneficial effect. However, it is also possible that individuals whose messages went undelivered faced different barriers to re-engagement than those whose messages were delivered. It was not possible to identify individuals in the SOC who would have received messages should they have been sent, and therefore a precise estimate of the effect of receipt of a message was not possible. As a result, we should be cautious about interpreting the per protocol analysis.

There was no difference in return to care for participants who received *framed* messages compared to *unframed* messages. Other studies have shown that fresh start dates can influence behaviour. For example, an observational study reported improved health behaviours such as searching for healthy diet information and gym attendance following a temporal landmark (beginning of week, month or year) [22] and an RCT of text messages encouraging COVID-19 vaccination reported that using fresh start dates increased vaccination rates [23]. Our study found that sending messages around a temporal landmark increased return to care, particularly for the Youth Day temporal landmark. It is possible that characteristics of temporal landmarks lead to differential impacts on behaviour. In our study, Mandela Day fell during school holidays, which may have affected healthcare utilization and therefore the impact of the message. However, highlighting the fresh start potential of the date did not further increase return. Similar to our study, an RCT that examined E-Mail reminders to improve adherence to chronic medication reported no improvement in clients who received a *framed* vs. an *unframed* E-Mail reminder sent on a birthday/new year [24]. More extensive testing of messages (frequency and timing), and the relevance of different temporal landmarks (beginning of year, birthday) could be useful in ensuring messaging interventions are more contextually appropriate and impactful.

We found that participants who had missed their visit by <6 months were more likely to return to care. Telephonic and in-person client tracing is part of the NDOH adherence guidelines [7], and studies have demonstrated the effectiveness of this intervention in re-engaging clients [10,21]. In people who have missed their visit by <6 months, the use of temporal landmarks and integration of text messaging could be considered for tracing programmes.

In multivariable logistic regression, receiving any text message was associated with increased odds of return to care for Youth Day and Mandela Day, although these associations did not reach statistical significance. Nonetheless, the trend is towards increased return to care with a text message. Longer ART duration and enrolment in differentiated ART models increased the odds of return. Longer ART duration has been reported as a predictor of reengagement in PWH in Mali [25]. Reduced odds of returning to care in those enrolled in priority clinics was an unexpected finding. We hypothesized that since these sites conduct more intensive client tracing, clients remaining out of care after tracing are less likely to return later. In addition, 80% of priority clinics are in a more urban area, which may lead to reduced odds of return due to higher mobility.

Strengths of this study include the RCT design while integrating the intervention into existing systems. The use of routine data to identify eligible individuals enabled inclusion of a large sample. This study had some limitations. Firstly, delays in capturing data on TIER.Net meant some data that determined eligibility were incomplete. This resulted in excluding participants later, which reduced the analysis sample size. Secondly, we relied solely on electronic data without medical record verification; therefore, our outcomes may be underestimated. Third, generalizability of our findings may be limited by the rural setting of this study. Furthermore, language and understanding of messages may differ based on context. Fourth, we did not have good routine data for individuals coming in and out of care, and the sample size calculation was based on data which did not adequately capture the number of people returning for HIV treatment. Fifth, Mandela Day and Youth Day results are not directly comparable because of the study design. The list of missed appointments was extracted once in June for both groups and the Youth Day messages were sent in June whilst the Mandela Day messages were sent in July. Therefore, a higher proportion of participants had returned to care before sending of text messages for Mandela Day (10.3%) compared to Youth Day (0.5%). These participants were excluded from the final analysis as shown in Fig. 1.

## Conclusion

Sending text messages around a fresh start date may result in higher return to HIV care compared to no text message, particularly for clients who have been out of care for less than six months. This study suggests the potential of text messages for re-engaging clients and could be considered as part of the package of client tracing within the first few months of a missed appointment. *Framed* text messages where temporal landmarks were highlighted did not improve return to care more than *unframed* text messages. Further studies are needed to understand the effect of framing messages to highlight the fresh start potential of temporal landmarks and the optimal timing of messages for maximum benefit.

## Acknowledgements

We would like to acknowledge the Limpopo Department of Health, including health clinic staff, and the Anova staff who managed the study procedures, in particular Basani Maluleke and Ntsetse Kgopong. We would also like to thank Dr Hengchen Dai for valuable input into the fresh start message design. We would also like to thank Laura Rossouw for assistance in reviewing the STATA code for the data analysis revisions.

Authors’ contributions: C.N., L.L., P.M., C.C.-M., B.M.-B., A.B., L.S., K.R. conceptualized the study. B.M. contributed to data curation. C.C.-M., P.M. and K.R. supervised study implementation and data analysis. C.N. conducted the data analysis. All authors contributed to manuscript writing and approved the final manuscript.

Financial support: This study was funded by the University of Pennsylvania via a Bill and Melinda Gates Foundation grant (INV008318). Anova Health Institute was co-funded by the US President's Emergency Plan for AIDS Relief (PEPFAR) through the United States Agency for International Development (USAID) under Cooperative Agreement number 72067418CA00023. L.L. was partially supported by the National Institute of Mental Health of the National Institutes of Health under grant number K01MH119923. The content is solely the responsibility of the authors and does not necessarily represent the official views of the funders or the United States Government.

Data sharing statement: Data for this study has been made publicly available and has been shared in a data repository. The link to access the data is https://doi.org/10.6084/m9.figshare.25272643.v1

### Conflicts of interest

There are no conflicts of interest to declare.

## Supplementary Material

Supplemental Digital Content
