# Supplementary material for: A randomized trial of ‘fresh start’ text messaging to improve return to care in people with HIV who missed appointments in South Africa
Source: AIDS. 2024 Jun 10;38(10):1579–88. doi: 10.1097/QAD.0000000000003939 (PMC11239091; doi:10.1097/QAD.0000000000003939)
Supplement: Supplemental Digital Content [file aids-38-1579-s001.docx]

**Supplementary Table 1:** SMS text messaging wording for Youth Day and Mandela Day temporal landmarks

| **Youth Day Thursday 16 June 2022** | | |
| --- | --- | --- |
| **Arm 2: *Unframed* text message** | **Message 1** | **Message 2** |
|  | Hello! This is a message from your clinic. We invite you to return to clinic soon, we missed you at your last appointment. Check in at Reception, we are ready to help. Welcome Back! Opt-out | Hi again from your clinic. We hope to see you very soon. Make a plan to visit us. Check in at Reception, we are ready to help. Welcome Back! Opt-out |
| **Arm 3: *Framed* text message** | **Message 1** | **Message 2** |
|  | Happy Youth Day for 16 June! We invite you to return to your clinic soon, we missed you at your last appointment. Your new beginning starts at Reception - we are ready to help. Welcome Back! Opt-out | Hope you enjoyed Youth Day! We hope to see you at the clinic very soon. Make a plan to visit that works for you. Your new beginning starts at Reception - we are ready to help. Welcome Back! Opt-out |
| **Mandela Day Monday 18 July 2022** | | |
| **Arm 2: *Unframed* text message** | **Message 1** | **Message 2** |
|  | Hello! This is a message from your clinic. We invite you to return to clinic soon, we missed you at your last appointment. Check in at Reception, we are ready to help. Welcome Back! Opt-out | Hi again from your clinic. We hope to see you very soon. Make a plan to visit us. Check in at Reception, we are ready to help. Welcome Back! Opt-out |
| **Arm 3: *Framed* text message** | **Message 1** | **Message 2** |
|  | Happy Mandela Day for 18 July! We invite you to return to your clinic soon, we missed you at your last appointment. Your new beginning starts at Reception - we are ready to help. Welcome Back! Opt-out | Hope you enjoyed Mandela Day! We hope to see you at the clinic very soon. Make a plan to visit that works for you. Your new beginning starts at Reception - we are ready to help. Welcome Back! Opt-out |
