# Supplementary material for: A randomized trial of ‘fresh start’ text messaging to improve return to care in people with HIV who missed appointments in South Africa
Source: AIDS. 2024 Jun 10;38(10):1579–88. doi: 10.1097/QAD.0000000000003939 (PMC11239091; doi:10.1097/QAD.0000000000003939)
Supplement: Supplemental Digital Content [file aids-38-1579-s002.docx]

**Supplementary Table 2:** Multivariable logistic regression of the association between predictor variables and ART visit outcome in participants randomized to no text message and any text message (*framed* and *unframed*) for Youth Day temporal landmark stratified by treatment interruption duration.

| **Treatment interruption <6 months-Youth Day (n=2211)** | | | | | | | **Treatment interruption ≥6 months-Youth Day (n=3643)** | | | | | |
| --- | --- | --- | --- | --- | --- | --- | --- | --- | --- | --- | --- | --- |
| **Variable** | **Unadjusted odds ratio** | **95%CI** | **P-value** | **Adjusted odds ratio** | **95%CI** | **P-value** | **Unadjusted odds ratio** | **95% CI** | **P-value** | **Adjusted odds ratio** | **95% CI** | **P-value** |
| **Text message arm** |  |  |  |  |  |  |  |  |  |  |  |  |
| No text message | **Ref** |  |  | **Ref** |  |  | **Ref** |  |  | **Ref** |  |  |
| Any text message* | 1.16 | 0.97-1.39 | 0.116 | 1.16 | 0.97-1.40 | 0.109 | 1.21 | 0.70-2.09 | 0.493 | 1.23 | 0.71-2.13 | 0.466 |
| **Age at randomisation (years)** |  |  |  |  |  |  |  |  |  |  |  |  |
| 18-24 | **Ref** |  |  | **Ref** |  |  | **Ref** |  |  | **Ref** |  |  |
| 25-49 | 1.31 | 0.89-1.92 | 0.171 | 1.27 | 0.86-1.89 | 0.231 | 0.52 | 0.20-1.33 | 0.171 | 0.43 | 0.17-1.13 | 0.088 |
| ≥50 | 2.19 | 1.44-3.34 | 0.000 | 1.89 | 1.22-2.93 | 0.004 | 0.66 | 0.22-1.97 | 0.460 | 0.54 | 0.17-1.65 | 0.276 |
| **Gender** |  |  |  |  |  |  |  |  |  |  |  |  |
| Male | **Ref** |  |  | **Ref** |  |  | **Ref** |  |  | **Ref** |  |  |
| Female | 0.96 | 0.79-1.16 | 0.677 | 0.99 | 0.81-1.20 | 0.894 | 1.67 | 0.85-3.25 | 0.134 | 1.71 | 0.86-3.38 | 0.124 |
| **ART duration (months)** |  |  |  |  |  |  |  |  |  |  |  |  |
| <6 | **Ref** |  |  | **Ref** |  |  | **Ref** |  |  | **Ref** |  |  |
| 6-12 | 2.28 | 1.44-3.62 | 0.000 | 2.38 | 1.49-3.79 | 0.000 | 2.88 | 0.83-10.01 | 0.095 | 2.83 | 0.81-9.86 | 0.102 |
| >12 | 3.34 | 2.43-4.59 | 0.000 | 2.97 | 2.13-4.13 | 0.000 | 6.11 | 2.42-15.48 | 0.000 | 5.62 | 2.18-14.52 | 0.000 |
| **Enrolled in differentiated care** |  |  |  |  |  |  |  |  |  |  |  |  |
| No | **Ref** |  |  | **Ref** |  |  | **Ref** |  |  | **Ref** |  |  |
| Yes | 1.49 | 1.23-1.80 | 0.000 | 1.14 | 0.93-1.41 | 0.201 | 2.23 | 1.11-4.48 | 0.024 | 1.66 | 0.80-3.44 | 0.174 |
| **Priority clinic** |  |  |  |  |  |  |  |  |  |  |  |  |
| No | **Ref** |  |  | **Ref** |  |  | **Ref** |  |  | **Ref** |  |  |
| Yes | 0.66 | 0.55-0.80 | 0.000 | 0.71 | 0.58-0.86 | 0.001 | 0.53 | 0.31-0.91 | 0.022 | 0.92 | 0.48-1.75 | 0.798 |
| **Sub-district** |  |  |  |  |  |  |  |  |  |  |  |  |
| Blouberg | **Ref** |  |  | **Ref** |  |  | **Ref** |  |  | **Ref** |  |  |
| Lepelle-Nkumpi | 1.17 | 0.84-1.62 | 0.357 | 1.15 | 0.82-1.60 | 0.422 | 0.98 | 0.46-2.10 | 0.965 | 0.92 | 0.43-1.99 | 0.837 |
| Molemole | 0.82 | 0.55-1.23 | 0.343 | 0.82 | 0.54-1.23 | 0.335 | 0.51 | 0.18-1.48 | 0.215 | 0.52 | 0.18-1.53 | 0.233 |
| Polokwane | 0.89 | 0.67-1.17 | 0.393 | 0.99 | 0.73-1.33 | 0.935 | 0.31 | 0.15-0.66 | 0.002 | 0.32 | 0.14-0.74 | 0.008 |

*Any text message- *framed* and *unframed* text message
