# Supplementary material for: A randomized trial of ‘fresh start’ text messaging to improve return to care in people with HIV who missed appointments in South Africa
Source: AIDS. 2024 Jun 10;38(10):1579–88. doi: 10.1097/QAD.0000000000003939 (PMC11239091; doi:10.1097/QAD.0000000000003939)
Supplement: Supplemental Digital Content [file aids-38-1579-s003.docx]

**Supplementary Table 3:** Multivariable logistic regression of the association between predictor variables and ART visit outcome in participants randomized to no text message and any text message (*framed* and *unframed*) for Mandela Day temporal landmark stratified by treatment interruption duration.

| **Treatment interruption <6 months - Mandela Day (n=1673)** | | | | | | | **Treatment interruption ≥6 months (n=3610)- Mandela Day** | | | | | |
| --- | --- | --- | --- | --- | --- | --- | --- | --- | --- | --- | --- | --- |
| **Variable** | **Unadjusted odds ratio** | **95% CI** | **P-value** | **Adjusted odds ratio** | **95% CI** | **P-value** | **Unadjusted odds ratio** | **95% CI** | **P-value** | **Adjusted odds ratio** | **95%CI** | **P-value** |
| **Text message arm** |  |  |  |  |  |  |  |  |  |  |  |  |
| No text message | **Ref** |  |  | **Ref** |  |  | **Ref** |  |  | **Ref** |  |  |
| Any text message* | 1.21 | 0.93-1.59 | 0.159 | 1.18 | 0.90-1.55 | 0.235 | 1.39 | 0.86-2.24 | 0.186 | 1.38 | 0.85-2.24 | 0.199 |
| **Age at randomisation (years)** |  |  |  |  |  |  |  |  |  |  |  |  |
| 18-24 | **Ref** |  |  | **Ref** |  |  | **Ref** |  |  | **Ref** |  |  |
| 25-49 | 1.02 | 0.62-1.66 | 0.948 | 0.90 | 0.55-1.49 | 0.695 | 0.40 | 0.19-0.85 | 0.017 | 0.35 | 0.16-0.76 | 0.008 |
| ≥50 | 1.36 | 0.79-2.36 | 0.268 | 1.11 | 0.63-1.98 | 0.711 | 0.43 | 0.17-1.09 | 0.076 | 0.32 | 0.12-0.85 | 0.021 |
| **Gender** |  |  |  |  |  |  |  |  |  |  |  |  |
| Male | **Ref** |  |  | **Ref** |  |  | **Ref** |  |  | **Ref** |  |  |
| Female | 1.38 | 1.03-1.87 | 0.033 | 1.41 | 1.03-1.92 | 0.031 | 0.85 | 0.51-1.41 | 0.521 | 0.82 | 0.49-1.38 | 0.461 |
| **ART duration (months)** |  |  |  |  |  |  |  |  |  |  |  |  |
| <6 | **Ref** |  |  | **Ref** |  |  | **Ref** |  |  | **Ref** |  |  |
| 6-12 | 1.04 | 0.48-2.25 | 0.921 | 0.99 | 0.46-2.14 | 0.977 | 0.73 | 0.24-2.19 | 0.574 | 0.75 | 0.25-2.27 | 0.613 |
| >12 | 2.44 | 1.57-3.79 | 0.000 | 1.86 | 1.16-2.97 | 0.010 | 2.24 | 1.27-3.96 | 0.005 | 2.22 | 1.23-4.01 | 0.008 |
| **Enrolled in differentiated care** |  |  |  |  |  |  |  |  |  |  |  |  |
| No | **Ref** |  |  | **Ref** |  |  | **Ref** |  |  | **Ref** |  |  |
| Yes | 1.99 | 1.52-2.62 | 0.000 | 1.67 | 1.23-2.26 | 0.001 | 1.42 | 0.70-2.88 | 0.334 | 1.18 | 0.56-2.49 | 0.671 |
| **Priority clinic** |  |  |  |  |  |  |  |  |  |  |  |  |
| No | **Ref** |  |  | **Ref** |  |  | **Ref** |  |  | **Ref** |  |  |
| Yes | 0.63 | 0.48-0.83 | 0.001 | 0.72 | 0.53-0.97 | 0.029 | 0.57 | 0.35-0.92 | 0.021 | 0.71 | 0.40-1.25 | 0.234 |
| **Sub-district** |  |  |  |  |  |  |  |  |  |  |  |  |
| Blouberg | **Ref** |  |  | **Ref** |  |  | **Ref** |  |  | **Ref** |  |  |
| Lepelle-Nkumpi | 1.15 | 0.72-1.84 | 0.567 | 1.07 | 0.66-1.72 | 0.794 | 1.34 | 0.61-2.95 | 0.463 | 1.41 | 0.64-3.11 | 0.395 |
| Molemole | 0.90 | 0.50-1.63 | 0.736 | 0.86 | 0.47-1.58 | 0.629 | 0.54 | 0.18-1.63 | 0.274 | 0.59 | 0.19-1.78 | 0.348 |
| Polokwane | 0.85 | 0.56-1.27 | 0.427 | 0.85 | 0.55-1.32 | 0.470 | 0.62 | 0.30-1.31 | 0.213 | 0.78 | 0.34-1.76 | 0.545 |

*Any text message- *Framed* and *unframed* text message
