# Supplementary material for: A randomized trial of ‘fresh start’ text messaging to improve return to care in people with HIV who missed appointments in South Africa
Source: AIDS. 2024 Jun 10;38(10):1579–88. doi: 10.1097/QAD.0000000000003939 (PMC11239091; doi:10.1097/QAD.0000000000003939)
Supplement: Supplemental Digital Content [file aids-38-1579-s004.docx]

**Supplementary Table 4:** Logistic regression of no text message compared to *unframed* and *framed* text messages and *framed* messages compared to *unframed* text messages (Youth Day & Mandela Day temporal landmark).

| **Youth Day temporal landmark** | | | | | | | **Mandela Day temporal landmark** | | | | | |
| --- | --- | --- | --- | --- | --- | --- | --- | --- | --- | --- | --- | --- |
| **Text message arm^a^** | **Unadjusted odds ratio** | **95% CI** | **P-value** | **Adjusted odds ratio** | **95%CI** | **P-value** | **Unadjusted odds ratios** | **95%CI** | **P-value** | **Adjusted odds ratio** | **95%CI** | **P-value** |
| No text message | **Ref** |  |  | **Ref** |  |  | **Ref** |  |  | **Ref** |  |  |
| *Unframed* text message | 1.25 | 1.04-1.51 | 0.016 | 1.27 | 1.03-1.57 | 0.024 | 1.28 | 0.98-1.69 | 0.072 | 1.22 | 0.92-1.63 | 0.167 |
| *Framed* text message | 1.04 | 0.86-1.26 | 0.709 | 1.07 | 0.86-1.33 | 0.525 | 1.26 | 0.96-1.65 | 0.104 | 1.22 | 0.92-1.63 | 0.175 |
| **Text message arm^b^** |  |  |  |  |  |  |  |  |  |  |  |  |
| *Unframed t*ext message | **Ref** |  |  | **Ref** |  |  | **Ref** |  |  | **Ref** |  |  |
| *Framed* text message | 0.83 | 0.67-1.02 | 0.081 | 0.85 | 0.67-1.09 | 0.191 | 0.98 | 0.72-1.32 | 0.886 | 0.98 | 0.71-1.35 | 0.898 |

1. All regression analysis adjusted for age at randomisation, gender, ART duration, treatment interruption stratification, enrollment into differentiated care, priority clinic, and sub-district.
2. All regression analysis adjusted for age at randomisation, gender, ART duration, treatment interruption stratification, enrollment into differentiated care, priority clinic, and sub-district.
